# Introduction of Heated Cow’s Milk Protein in Challenge-Proven Cow’s Milk Allergic Children: The iAGE Study

**DOI:** 10.3390/nu14030629

**Published:** 2022-01-31

**Authors:** Nicolette W. de Jong, Marloes E. van Splunter, Joyce A. M. Emons, Kasper A. Hettinga, Roy Gerth van Wijk, Harry J. Wichers, Huub F. J. Savelkoul, Aline B. Sprikkelman, R. J. Joost van Neerven, Liu Liu, Gerbrich van der Meulen, Irene Herpertz, Yvonne C. M. Duijvestijn, Mijke Breukels, Marianne I. Brouwer, Jaap Schilperoord, Olga van Doorn, Berber Vlieg-Boerstra, Jochum van den Berg, Linette Pellis, Severina Terlouw, Astrid I. Hendriks, Marco W. J. Schreurs, Frank E. van Boven, Nicolette J. T. Arends

**Affiliations:** 1 Department of Internal Medicine, Section of Allergology & Clinical Immunology, Erasmus MC, University Medical Centre Rotterdam, 3015GD Rotterdam, The Netherlands; m.e.vansplunter@erasmusmc.nl (M.E.v.S.); r.gerthvanwijk@erasmusmc.nl (R.G.v.W.); s.terlouw@erasmusmc.nl (S.T.); f.boven@erasmusmc.nl (F.E.v.B.); 2 Depertment of Peadiatric Allergology, Sophia Children Hospital, Erasmus MC, University Medical Centre Rotterdam, 3015GD Rotterdam, The Netherlands; j.a.m.emons@erasmusmc.nl (J.A.M.E.); a.hendriks-vantoor@erasmusmc.nl (A.I.H.); n.arends@erasmusmc.nl (N.J.T.A.); 3 Food Quality & Design Group, Wageningen University & Research Centre, 6708PB Wageningen, The Netherlands; kasper.hettinga@wur.nl (K.A.H.); harry.wichers@wur.nl (H.J.W.); 4 Cell Biology & Immunology Group, Wageningen University & Research Centre, 6708PB Wageningen, The Netherlands; huub.savelkoul@wur.nl (H.F.J.S.); joost.vanneerven@wur.nl (R.J.J.v.N.); 5 Department of Peadiatric Pulmonology & Allergology, UMCG, University Medical Center Groningen, 9713GZ Groningen, The Netherlands; a.b.sprikkelman@umcg.nl; 6 University Medical Center Groningen, GRIAC Research Institute, University of Groningen, 9713GZ Groningen, The Netherlands; 7 FrieslandCampina, 3811LP Amersfoort, The Netherlands; 8 Department of Rheumatoloy, Leiden University, 2311BD Leiden, The Netherlands; l.liu@outlook.com; 9 Department of Peadiatrics, Martini Hospital, 9728NT Groningen, The Netherlands; g.vander.meulen@mzh.nl; 10 Paediatric Allegology Center, VieCuri Medisch Centrum, 5912BL Venlo, The Netherlands; I.Herpertz@mzh.nl; 11 Department of Peadiatrics, Noordwest Ziekenhuisgroep, 1815JD Alkmaar, The Netherlands; y.c.m.duijvestijn@nwz.nl; 12 Deparment of Peadiatrics, Elkerliek, 5707HA Helmond, The Netherlands; mbreukels@elkerliek.nl; 13 Department of Peadiatrics, Canisius Wilhelmina Ziekenhuis, 6532SZ Nijmegen, The Netherlands; m.l.brouwer@cwz.nl; 14 Department of Peadiatrics, OLVG Hospital, 1091AC Amsterdam, The Netherlands; j.v.schilperoort@olvg.nl (J.S.); o.vandoorn@olvg.nl (O.v.D.); b.vlieg-boerstra@olvg.nl (B.V.-B.); 15 Department of Peadiatrics, Zuyderland MC, 6419PC Heerlen, The Netherlands; joc.vandenberg@zuyderland.nl; 16 Ausnutria B.V., 8025BM Zwolle, The Netherlands; Linette.Pellis@ausnutria.nl; 17 Department of Immunology, Erasmus MC, University Medical Centre Rotterdam, 3015GD Rotterdam, The Netherlands; m.schreurs@erasmusmc.nl

**Keywords:** allergy, baked milk, cow’s milk, tolerance

## Abstract

The introduction of baked milk products in cow’s milk (CM) allergic children has previously been shown to accelerate induction tolerance in a selected group of children. However, there is no standardized baked milk product on the market. Recently, a new standardized, heated and glycated cow’s milk protein (HP) product was developed. The aim of this study was to measure safety and tolerability of a new, well characterized heated CM protein (HP) product in cow’s milk allergic (CMA) children between the age of 3 and 36 months. The children were recruited from seven clinics throughout The Netherlands. The HP product was introduced in six incremental doses under clinical supervision. Symptoms were registered after introduction of the HP product. Several questionnaires were filled out by parents of the children. Skin prick tests were performed with CM and HP product, sIgE to CM and α-lactalbumin (Bos d4), β-lactoglobulin (Bos d5), serum albumin (Bos d 6), lactoferrin (Bos d7) and casein (Bos d8). Whereas 72% percent (18 out of 25) of the children tolerated the HP product, seven children experienced adverse events. Risk factors for intolerance to the HP product were higher skin prick test (SPT) histamine equivalent index (HEP) results with CM and the HP product, higher specific IgE levels against Bos d4 and Bos d8 levels and Bos d5 levels. In conclusion, the HP product was tolerated by 72% of the CM allergic children. Outcomes of SPT with CM and the HP product, as well as values of sIgE against caseins, α-lactalbumin, and β-lactoglobulin may predict the tolerability of the HP product. Larger studies are needed to confirm these conclusions.

## 1. Introduction

The prevalence of food allergies varies considerably, depending on self-reported food allergy (FA) sensitization to food allergens or confirmed food allergy by an open food challenge (OFC) test, or, preferably, a double-blind, placebo-controlled food challenge (DBPCFC) test. In Europe, the prevalence of challenge-proven/confirmed cow’s milk allergy (CMA) in young children (< age 3 year) varies between 0.35 and 2.0% [1,2].

About 70% of CMA children reach clinical tolerance to milk proteins before the age of two years, and 60% before the age of three years, indicating transient CMA [3]. Factors that are most predictive for spontaneous resolution of CMA are: low milk-specific IgE level (<2 kU/L), small-wheal CM skin prick test (SPT) (<5 mm) and absence of (or mild) atopic dermatitis (AD) [4].

Several studies have reported high percentages (59–81%) of tolerance to baked milk in CMA children [4,5,6,7]. Children not tolerant towards baked milk products have an increased risk of developing a persistent CMA compared to children tolerant of baked milk (relative hazard ratio: 0.28 vs. 4.1) [3].

CMA children are mostly sensitized (presence of specific IgE to cow’s milk proteins) to multiple cow’s milk proteins. Sensitization can be found to caseins, including αs1-, αs2-, β- and kappa casein (together constituting 80% of cow’s milk proteins (CMP)), and/or to whey proteins, such as α-lactalbumin (Bos d4) and β-lactoglobulin (Bos d5) [8,9,10]. To a lesser extent, sIgE against bovine serum albumin and other whey proteins is also found [8]. In particular, high levels of sIgE against αs1-, β-casein, Bos d4 and Bos d5 are associated with persistent CMA [11,12,13]. In addition to IgE-mediated cow’s milk allergy, a substantial part of the children has non-IgE-mediated CMA, resulting in delayed-type reactions (>2 h) lacking the typical IgE-mediated symptoms (urticaria, angioedema, respiratory and/or gastro-intestinal symptoms or anaphylaxis) [1,13,14]. About 90% of patients with CMA also react to goat’s milk, due to high protein homology (95%) and high protein identity (>84%) [15].

Consumption of baked milk products in CMA children appeared to accelerate tolerance induction in a selected group of children [6,16]. Moreover, a recent meta-analysis showed that most studies were observational, lacking an appropriate control group [17]. Processing milk proteins changes their immunogenic and allergenic properties and can lead to both allergy and the induction of tolerance [18,19]. For example, when milk sugar lactose and CMP are heated together, glycation takes place, causing sugars to be linked to the free amino groups of amino acids [20]. This can lead to the formation of advanced glycation end products (AGEs), which can mask existing epitopes but can also create new immunogenic structures [20,21,22].

In making baked milk products for previous studies on tolerability, both the form of the product (e.g., cake, bread, or cheese on pizza), and the precise heating process were found to be highly variable. Especially when baking products such as cakes, the internal temperature of the product may strongly differ from the surface temperature, leading to different heat-induced protein modifications throughout the product, which may even leave some of the CMP intact. For better understanding of the effect of baked milk on CMA, a standardized, heated and glycated CMP product should be produced, in which the extent of heat-induced modifications, including glycation, have been well characterized [6,16,23,24,25].

The aim of this study was to measure safety and tolerability of a new, well characterized heated cm protein (HP) product in CMA children between the age of 3 and 36 months.

## 2. Materials and Methods

### 2.1. Study Population

This study was performed in seven hospitals throughout The Netherlands: two university hospitals and five large referral hospitals with expertise in paediatric allergy.

Children between three months and three years of age with cow’s milk allergy diagnosed by a doctor (paediatric allergist from participating centres) were approached for participation in the study.

After inclusion, a DBPCFC or OFC with cow’s milk was performed. Children with a recent (<6 months) positive CM challenge or a previously severe allergic reaction after CM consumption reported by the participating clinicians did not undergo a cow’s milk challenge. Sensitization to cow’s milk was measured in SPT and sIgE. Parents filled out several questionnaires.

During a whole-day visit at the outpatient clinic, the HP product was introduced to the diet of the child. Either parents or legal guardians had to understand the Dutch language and signed informed consent. The medical ethical committee of each participating centre approved the study. (NL61774.078.17).

### 2.2. Double-Blind, Placebo Controlled Food challenge (DBPCFC) with CM

A DBPCFC with CM was performed according to the guidelines. Children consuming extensively hydrolysed formula or formula based on amino acids were challenged using standardized kits. Children consuming breastmilk or alternative milk (e.g., soy milk or rice milk) were challenged using the matrix they consumed. The dosage of CMP in the challenge test remained standard (1-3-10-30-100-300 mg, etc.; Table 1). Symptoms (both subjective and objective) were scored according PRACTALL [26,27].

### 2.3. Study Product: Heated Glycated CM Protein (HP)

The HP product was produced by FrieslandCampina (Amersfoort, The Netherlands) and was a powdered product that contained a mixture of whey (20%) and casein protein (80%). The HP product was treated at ultra-high temperature (UHT) (120 °C) for 20 min, spray-dried and subsequently canned. The cans were stored at 60 °C for 14 days, resulting in glycation of the CMPs. The products and procedures for making the AGE products were judged and approved by the Quality assurance/Quality Control (QA/QC) department of FrieslandCampina to be compliant with IFT guidelines. The amount of carboxymethyllysine (CML) (a measure for glycation) in regular infant formula is 28–81 ng CML/mg protein [28]. CML analysis of the new HP product showed a result of 300 ng CML/mg protein, which is comparable to evaporated milk.

### 2.4. Introduction of HP Product

The HP product was introduced in incremental doses in 30 min intervals (10-30-100-300 mg etc. Table 1) [27]. The HP product was dissolved in the participant’s individual daily milk formula (5% of total protein intake/day). The symptoms were recorded in a database/chart according to PRACTALL and scored on severity retrospectively by two independent paediatricians [29]. Serious adverse events (SAEs) and adverse events (AEs) were reported to a data safety monitoring board (DSMB).

### 2.5. Skin Prick Tests (SPT)

An SPT was performed by applying a drop of skimmed CM (FrieslandCampina), goat’s milk (Ausnutria B.V., Zwolle, The Netherlands), the HP study product, a positive (histamine) and a negative (PBS) control. Subsequently, the surface area was measured with an area scanner and compared with the positive control, which gives the histamine equivalent prick (HEP) index score as described [30]. No threshold values have yet been defined for the skin prick test (SPT)–HEP index values for cow’s milk allergy. SPTs were considered positive at values > 3 mmØ [31].

### 2.6. Serum Collection and sIgE Measurements

Blood samples were collected using either a finger prick (age < 6 months) or a vena puncture (≥6 months of age). Serum was collected and stored at −20 °C. ISAC (81-1020-01, Thermo Fisher Scientific B.V, Breda, The Netherlands) was used to identify specific sIgE against CM protein. In addition, sIgE against total CMP was performed using Immunocap (f2) (14-4112-01, Thermo Fisher Scientific B.V., Breda, The Netherlands).

The following specific recombinant allergens were measured by both ISAC and ImmunoCap: α-lactalbumin (Bos d4), β-lactoglobulin (Bos d5), bovine serum albumin (Bos d6), (immunoglobulins/lactoferrin (Bos d7) and whole casein (Bos d8) [32].

### 2.7. Questionnaires

Validated questionnaires, as used in the “Generation R” study, were used to assess the medical history of mother, father and child [33]. Data collected included date of birth, sex, race, ethnicity, height, weight and relevant medical history. To measure eczema severity, POEM and eczema area and severity index (EASI) scores were collected [34]. Furthermore, a questionnaire specifically designed for this study was used to gather information from the parents about the child’s current situation in relation to, e.g., atopy, dairy consumption, type of formula, feeding or breastfeeding, introduction of other (solid) food and type of symptoms. The Food allergy quality of life questionnaire (FAQLQ) was implemented according to Velde et al. [35].

### 2.8. Open Clinica Database

All patient-related information of this study (Case report forms (CRFs)) is kept blinded at Erasmus MC, and data were digitalized in an Open Clinica (OpenClinica, LCC, Waltham, MA, USA) study database (version 3.12.2).

### 2.9. Statistical Analysis

This study was originally part of a large long-term clinical trial to test tolerance-induction of the HP product. As the trial was hampered by a low inclusion rate, we decided to perform a small exploratory study with the available patients, focusing on measuring safety and tolerability of the product. For further analyses focused on this aim, the Bayesian approach is the recommended method for evaluating small samples [36]. The Bayes factor (BF) produces the likelihood ratio of the alternative hypothesis (H1) (difference between groups) and the null hypothesis (H0) (no difference between groups) [37]. Evidence for the alternative hypothesis (H1) was set as BF > 3 (moderate), BF > 10 (strong), BF > 30 (very strong) and BF > 100 (extreme), and evidence for the null hypothesis (H0) was set as BF < 1 [37]. BF was calculated for proportions of positive sIgE, contingency tables and two-sample designs of SPT-HEP indexes by the Bayes factor package in R, version 4.0.4/ (https://CRAN.R-project.org/package=BayesFactor, accessed on 15 February 2021). Priors in the proportions were set to low, mediocre, high or unknown probability. In the two-sample designs, the prior distribution was set to a Cauchy with rscale = 0.707 [38].

## 3. Results

In total, 25 CMA children participated in this study: 9 girls and 16 boys, with a mean age of 14.5 months (range: 6–37 months). A total of 18 children were tolerant to the HP product (HPt group), and seven children developed an allergic reaction to the HP product (HPr group). No differences were found in baseline characteristics (e.g., eczema, rhinitis and asthma) between both groups, as shown in Table 2.

Baseline measurements comparing differences in sensitization (SPT, sIgE) to CM and CM components are shown in Table 3. In the HPt group, 10/17 (59%) children, and in the HPr group 4/5 (80%) children, had a positive SPT (>3 mmØ) for CM (BF 0.6). Specific serum IgE to CM was positive in 10/15 (67%) children in the HPt group and in 4/5 (80%) children in the HPr group (BF 2.4). Most children were sensitized both in SPT and sIgE to whole CM, but in some cases, only one was positive. Results show that the group does not contain non-IgE-mediated CMA children, although in some individual cases, symptoms in DBPCFC occurred > 2 h after the challenge. The HPt group showed lower sIgE levels to Bos d4 (BF 6,2) and Bos d8 (BF 17,8) in comparison with the HPr group. On the contrary, the HPr group showed lower Bos d5 sIgE levels (BF 6,2). The SPT with HP product was found in only half of all children who tested positive (11/22): six (35%) in the HPt group and four (80%) in the HPr group (BF 6.5). SIgE values measured with ISAC against house dust mite, grass pollen and birch pollen were negative in all children.

No differences were observed for characteristics of the parents, households and the background of children, e.g., familial atopic diseases, between the HPt group and HPr group. (Table 4) The use of antibiotics in the children was higher in the HPt group versus the HPr group (BF 9.4). The percentage of children going to a day-care facility was higher in the HPt group versus the HPr group (BF 3.1), and breastfeeding (ever) was higher in the HPt group versus the HPr group (BF 29.1). More children in the HPt group received CM formula feeding in the first week of life compared to the HPr group (61% vs. 28.6%, respectively).

Table 5 shows the adverse events in the HPr group (*n* = 7) during introduction of the HP product. Ref. [31] One patient experienced an SAE (ID: 111002), which started 15 min after the second step (3 mg CM protein). Six patients experienced AEs. An overview of the category and type of symptoms developed at a certain step of HP product is given in Supplementary Table S1.

No differences were found in baseline CM challenge (DBPCFC or OFC) between HPt and HPr groups. In total, 17/25 challenges were double blind. In one child, no challenge was performed due to two anaphylactic reactions to CM in recent history. In some individual cases, symptoms in DBPCFC occurred > 2 h after the challenge. The symptoms that occurred in these individuals were skin disorders and vomiting. Epinephrine auto-injectors had to be used three times in the HPt group, and no epinephrine was administered in the HPr group (Supplementary Table S2).

In the FAQLQ, no differences were found between groups, except one: we found a lower parental perception (<2 = very small chance) on appropriate response by others to allergic reactions in their child in the HPr group (HPt group mean: 2.28; range 0–6; HPr group mean: 1.5; range 1–2) [6] (Supplementary Table S3).

## 4. Discussion

This study aimed to investigate the safety and tolerability of a new heated and glycated cow’s milk protein product, the HP product. This HP product aimed to mimic and to have tolerance-inducing capacities similar to those of “baked milk” products, with well-defined production methods, e.g., exact heating time and temperature during glycation process, in order to achieve a predetermined glycation level.

Due to low inclusion numbers, the primary aim to study tolerance-inducing capacity of the HP product could not be reached, and planned statistical analyses could not be performed. Nevertheless, in alternative analyses (e.g., Bayes factor) differences between HPt and HPr children could be analysed and were, in some cases, significantly high and should be considered exploratory.

Low inclusion numbers in intervention studies in children with cow’s milk allergy have been described previously. Many studies on specific food allergy in children are underpowered, according to a recent Cochrane database systematic review on effects of eHF use in CMA children [38]. Reasons for the low inclusion numbers in the current study were less motivated parents due to the many planned hospital visits with the child during the study, following a strictly cow’s-milk-free diet and low numbers of positive cow’s milk DBPCFC in children with suspected CMA. The latter is not surprising, as recent research by Vlieg et al. in The Netherlands reported a considerable percentage of overdiagnoses of CMA in children (66%) when children did not react in a DBPCFC [39].

Eighteen out of the 25 included CMA children (72%) were tolerant of the HP product (HPt group). Sensitization patterns differed between the two groups. Children in the HPr group showed a higher sensitization profile. This might be one of the reasons for allergic reactions to the HP product [40]. It is known from literature that, in contrast to whey proteins, caseins do not denature and aggregate upon heat treatment but can be glycated [41]. These lesser changes due to heat treatment of the caseins may explain the intolerance of the HP product in this group. Sensitization to caseins was much higher in the HPr group (60%) in comparison with the HPt group (only one child; 7%).

At the same time, the HPr group had clearly lower sIgE against β-lactoglobulin (Bos d5). β-lactoglobulin can be denatured and aggregated after heating, but this HPr group was borderline sIgE-positive (0.42 ISU) for β-lactoglobulin, so that could not affect the tolerability to the heated cow’s milk protein in this group. Sensitization level in SPT to goat’s milk and CM was comparable between groups (BF 0.6 and 0.5, respectively). Apparently, the HP product cannot be tolerated in a substantial number of children with goat’s milk sensitization.

Regarding baseline characteristics, in the HPr group, the use of antibiotics (BF: 9.4) and attendance at day care was lower (BF 3.1) Furthermore, all children in the HPr group received breast feeding (ever), whereas only half of the children in the HPt group did (BF 29.1). When children attend day care to a lesser extent, a lower use of antibiotics seems logical, as infections occur less often in “no-day-care” children [42], and longer breastfeeding is easier in practical terms. Although breastfeeding has many benefits, it does not reduce the risk of CMA [43].

Combining all results of this study, we hypothesize that a possible cause of intolerance of the HP product (HPr group) lies in an overall lower general exposure to allergens (less day care and infections) in the first months of life, as previously described by McGowan et al. [44], and to milk allergens in specific.

Seventy-two per cent of the patients could tolerate the HP product. This is comparable with data from the “baked milk studies” [4,5,6,7,16]. In a more recent study by Agyemang et al. among 84 children, 72% were tolerant to muffins containing CM [40]. Furthermore, the HPt group showed > 90% negative sIgE to caseins, most likely representing a group with transient CMA more likely to tolerate baked milk products [8].

In the current study, the safety profile of the HP product was found to be comparable with larger studies with baked milk challenges [45].

The tolerance-inducing effects of baked milk products are described in many studies, but the effect of heating and glycation on tolerance-inducing effects is only sparsely investigated. With this new HP product, we tried to mimic “baked milk” products, while standardizing its characteristics and production. This is, as far as we could find in the literature, the first well described “baked milk” product. The powder can be easily added to the daily formula of very young CMA children, who, in some cases, might not yet be able to consume baked products, e.g., cake. However, the introduction can cause mild to severe allergic reactions and should therefore be supervised by a clinician. Sensitization profiles to CM can be useful to pre-select children who will most likely tolerate the HP product. Further trials with this promising new HP product should be performed in larger groups of children to the measure the tolerance-inducing capacity.

In summary, a new HP product was found to be safe and was tolerated by 72% of challenge-proven CMA children. Outcomes of SPT with CM and the HP product, as well as values of sIgE against caseins, α-lactalbumin and β-lactoglobulin, may predict the tolerability of the HP product. Larger studies are needed to confirm these conclusions.

## Figures and Tables

**Table 1:** Dosages in double-blind, placebo-controlled CM challenge and HP product introduction, as well as cumulative dosage.

	CM DBPCFC	CM DBPCFC		Open Introduction	Open Introduction
Step	CM protein (mg)	Cumulative dosage (mg)	Step	HP product (mg)	Cumulative dosage (mg)
1	1	1		-	-
2	3	4		-	-
3	10	14	1	10	10
4	30	44	2	30	40
5	100	144	3	100	140
6	300	444	4	300	440
7	1000	1444	5	1000	1440
8	Age-dependent	Age-dependent	6	2000	3440

CM: cow’s milk; DBPCFC: double-blind, placebo-controlled food challenge; mg: milligrams; HP product: heated cow’s milk product.

**Table 2:** Baseline characteristics of the HP-tolerant (HPt) and HP-reactive (HPr) children.

		HPt Children (*n* = 18)				HPr Children (*n* = 7)			
		Mean	Range	N Pos (T)	%	Mean	Range	N Pos (T)	%
	Age (months)	14.6	(6.5–22.5)	18		13.3	(6.1–37)	7	
	Gender: F(tot)			6 (18)	33.3			3 (7)	42.9
Atopy *	Eczema			11 (18)	61.1			3 (6)	50.0
	EASI			5 (18)	27.8			2 (6)	33.3
	POEM			10 (18)	55.6			3 (6)	50.0
	Rhinitis			4 (18)	22.2			0/6	0
	Asthma-like symptoms			3 (18)	16.7			1 (6)	16.6
	Asthma + medication			2 (18)	11.1			0 (6)	0
Exclusively breastfed	Period, (months)	5.2	(1–9)	9 (18)	50%	3.2	(2–7)	7 (7)	100%
Formula use at inclusion visit	eHF			13 (18)	72%			5 (7)	71%
	AA			5 (18)	27%			2 (7)	29%
Multiple food allergy	Egg, peanut and/or nuts			4 (18)	22%			0 (7)	0%

HPt: HP-product-tolerant; HPr: HP-product-reactive; *: parent reported; EASI: eczema area and severity index, T: totals; eHF: extra hydrolysed formula; AA: amino acid formula.

**Table 3:** Baseline sensitization profiles of the HP-product-tolerant (HPt) and HP-reactive (HPr) children.

		HPt Children (*n* = 18)				HPr Children (*n* = 7)				BF
		Mean	Range	N Pos (T)	%	Mean	Range	N Pos (T)	%	
SPT *	CM	0.72	(0–2.98)	10 (17)	58.8	1.17	(0–1.72)	4 (5)	80	0.6
	Goat’s milk	0.54	(0–4.23)	6 (15)	40	0.86	(0.22–1.16)	4 (4)	100	0.5
	HP product	0.23	(0–1.58)	6 (17)	35.3	1.06	(0–2.33)	4 (5)	80	6.5
sIgE	CM (kU/L)	3.08	(0–17.2)	10 (15)	66.7	14.93	(0.01–49.6)	4 (5)	80	2.4
	α-lactalbumin Bos d4 (ISU)	0.41	(0–3.36)	5 (15)	33.3	1.48	(0–5.45)	4 (5)	80	6.2
	β-lactoglobulin Bos d5 (ISU)	1.47	(0–10.7)	5 (15)	33.3	0.42	(0–0.8)	4 (5)	80	6.2
	Bovine serum albumin Bos d6 (ISU)	0.16	(0–0.79)	4 (15)	26.7	0.5	(0–2.5)	1 (5)	20	1.3
	Casein Bos d8 (ISU)	0.09	(0–1)	1 (15)	6.7	0.74	(0–2.3)	3 (5)	60	17.8
	Lactoferrin (ISU) Bos d7 (ISU)	-	-	0/15	0	-	-	0 (5)	0	NA

HPt: HP-product-tolerant; HPr: HP-product-reactive; *: HEP index; CM: cow’s milk; BF: Bayes factor theorem; H0 two values/means are equal; H1 two values/means are not equal. BF < 1 = H0 most likely; BF ≥ 3 = H1 most likely.

**Table 4:** Characteristics of the parents, households and the background of children.

		HPt Children (*n* = 18)		HPr Children (*n* = 7)		BF	Prior Chance
		N Pos (T)	%	N Pos (T)	%		
Atopy (parents)	Mother	13 (18)	72.2	5 (7)	71.4	1.1	mediocre
	Father	11 (18)	61.1	4 (7)	57.1	1.3	mediocre
	Both parents	7 (18)	38.9	2 (7)	28.5	2.6	low
	Both parents not	1 (18)	5.5	0 (7)	0	NA	NA
Background (child)	Breastfeeding (ever)	9 (18)	50	7 (7)	100	29.1	high
	Antibiotics use (child)	12 (18)	66.7	2 (7)	28.5	9.4	low
**Pregnancy Mother**	Antibiotics use	12 (18)	17.6	0(7)	0	1.3	mediocre
	Folic acid	16 (17)	94.1	6 (7)	85.7	0.4	unknown
	Vitamin D suppl.	2 (16)	12.5	1 (7)	14.3	1.8	low
	Ω-3 fatty acid suppl.	2 (16)	12.5	1 (7)	14.3	1.8	low
	fish oil capsules	0 (15)	0	1 (7)	14.3	0.6	unknown
	multivitamin suppl.	13/17	76.5	4 (7)	57.1	0.7	unknown
Exposure to smoke	During pregnancy *	4 (17)	23.5	2 (7)	28.6	2.3	low
	Current smoking	1 (17)	5.9	0 (7)	0	0.7	unknown
Pet keeping	Currently	1 (17)	58.8	4 (7)	57.1	1.3	mediocre
Other	Day care	11 (18)	61.1	2 (7)	28.6	3.1	mediocre

HPt: HP-product-tolerant; HPr: HP-product-reactive; *: passive smoking + current smoking; *n*: number; BF = Bayes factor: H0 two values/means are equal; H1 two values/means are not equal. BF < 1 = H0 most likely; BF ≥ 3 H1 most likely.

**Table 5:** Serious adverse events and adverse events (SAE/AE).

Patient ID	Age Months/ M/F	Allergic Reactions at Step	Minutes after Intake	Medication, According to National Anaphylaxis Protocol	Stopped at Dose/Outcome *	SAE/AE Sampson Scale
111002	13/M	Step 2:	Step 2:	Step 2:	Dose: 2/ After 5 h released from hospital	SAE 4
		Eczema lips (15′ diminished)	2 min	Adrenaline auto-injector (0.15 mg), Xyzal suspension (2.5 mg), dexamethason (4 mg)		
		Step 2 repeated: Stridor, cough, crying	Step 2 repeated: 15 min			
555004	6/F	Step 6:	Step 6:	None	Full challenge/ <24 h	AE/2
		Eczema feet, back, belly;	15 min;			
		vomiting, itch, eczema face, diarrhea	6–9 h			
888004	37/M	Dry cough, stridor	Step 4: 5–10 min	Step 4:	Dose: 4/ After 2 h released from hospital	AE/4
				Aerius suspension (2.5 mL)		
888005	8/M	Step	Step	Step 5:	Dose: 5/ After 2 h released from hospital	AE/3
		1: Sneezing, erythema chin	1 repeated: 5 min	Aerius-suspension (2x 2.2 mL)		
		5: Cough, redness face, nausea	5: 10–15 min			
999001	11/M	Step:	Step 3: 25 min	Step 3: Aerius suspension (2.5 mL) and prednisone	Dose: 3/ 15 min no more wheezing	AE/3
		2: increased eczema				
		3: Sneezing, cough, runny nose, increased eczema, wheezing				
999002	10/F	Step:	Step:	none	Dose: 6/ Not specified	AE/3
		4: Sneezing, cough, starting urticaria	4: 15–20 min			
		5: Runny nose, redness face, urticaria	5: directly			
		6: (after pause): urticaria	6: after 10 min			
999004	10/F	Step:	Step:	Step 2: Aerius suspension (2.5 mL) and 1.6 mL prednisone	Dose: 2/ After 4 h released from hospital	AE/2

Abbreviations: Step 1 (10 mg), step 2 (30 mg), step 3 (100 mg), step 4 (300 mg), step 5 (1000 mg), step 6 (3000 mg). * Hours until symptoms disappeared; SAE: serious adverse events; AE: adverse events; M/F: male/female; mL: millilitres; min: minutes.

## Supplementary Materials

The following are available online at https://www.mdpi.com/article/10.3390/nu14030629/s1, Table S1: Characteristics of patients with a reaction during introduction of the heated cow’s milk protein study product. Table S2: Baseline CM food challenge; comparison between HP-product-tolerant children (HPt) and HP-product-reactive (HPr) in developed symptoms and emergency medication. Table S3: Results FAQ-LQ (D-Q1) parents of children in HPt group and HPr group.
