# Supplementary material for: Introduction of Heated Cow’s Milk Protein in Challenge-Proven Cow’s Milk Allergic Children: The iAGE Study
Source: Nutrients. 2022 Jan 31;14(3):629. doi: 10.3390/nu14030629 (PMC8838309; doi:10.3390/nu14030629)
Supplement: Supplementary file 1 [file nutrients-14-00629-s001.zip › nutrients-1550964-supplementary.pdf]

Supplementary Tables S1, S2 & S3 iAGE study NW de Jong.

Suppl. Table S1: Characteristics of patients with a reaction during introduction of the Heated Cow's milk Protein study product

	HPr children (7)			
	Mean	(Min-max)	N=	%
Eliciting step symptoms	2.4	(1-4)	6/7	8.7
Last step given	4.1	(2-6)	5/7	71.4
Symptoms observed:				
Skin symptoms				
Category 1			6	85.7
Category 2			3	4.9
Category 3			1	1.3
Airway symptoms				
Category 1			5	71.4
Category 2			5	71.4
Category 3			2	28.6
Eyes				
Category 1			0	0
Category 2			1	1.3
Category 3			0	0
Gastrointestinal symptoms				
Category 1			0	0
Category 2			2	28.6
Category 3			1	14.3
Other symptoms*				
Category 1			1	14.3

Category 2			0	0
Category 3			0	0
Emergency medication				71.4
Antihistamine (N=)			5	
Corticosteroids (N=)			3	
Adrenaline (N=)			1	

HPr: HP product reactive. Symptoms (both subjective and objective) were scored according PRACTAL: category 1: mild; category 2: moderate, category 3: severe.* Other symptoms e.g., crying

Suppl Table S2: Baseline CM food challenge; comparison between HP product tolerant children (HPt) and HP product reactive (HPr) in developed symptoms and emergency medication

	HPt children (18)				HPr children (7)				BF
	Mean	(Min-max)	N=	%	Mean	(Min-max)	N=	%	
Eliciting step	4.1	(1-8)	15/15		2.9	(1-8)	7		0.5
Last step given	7.5	(4-8)			6.8	(3-8)			0.9
Skin symptoms									
Category 1			4	26.7			6	85.7	
Category 2			11	73.3			5	71.4	
Category 3			3	20			1	14.3	
Airway S.									
Category 1			0	0			4	57.1	
Category 2			2	13.3			3	42.9	
Category 3			1	6.7			2	28.6	
Eyes									
Category 1			0	0			0	0	
Category 2			1	6.7			0	0	

Category 3			1	6.7			0	0	
GI S									
Category 1			0	0			1	14.3	
Category 2			0	0			1	14.3	
Category 3			0	0			7	0	
Other symptoms*									
Category 1			6	40.0			3	42.9	
Category 2			4	26.7			0	0	
Category 3			1	6.7			1	14.3	
Emergency medication			9	60.0%			4	57.1%	
oral antihistamine			9X				2X		
Corticosteroids			4X				2X		
Adrenaline			3X				0X		

HPt : heated protein product tolerant; HPr: heated protein product reactive. GI: Gastro-intestinal; AH: Anti=histamine; BF= Bayes factor: H0 two values/means are equal; H1 two values/means are not equal. BF<1= H0 most likely; BF≥3 H1 most likely. Symptoms (both subjective and objective) were scored according PRACTAL: category 1: mild; category 2: moderate, category 3: severe. * Other symptoms: e.g., restless, crying, refuse to drink

Supplementary Table S3: Results FAQ-LQ (D-Q1) parents of children in HPt group and HPr group.

FAQ-LQ (D-Q1) How big is the chance according to the parents that their child:	HPt children (N=18)	HPr children (N=7)
	Mean (range)	Mean (range)
Q1 Accidentally eats something he/she is allergic to	2.22 (1-4)	2.5 (1-4)
Q2 Gets a severe allergic reaction due to FA	3.5 (1-6)	3.33 (2-6)
Q3 Dies due to FA	1 (0-4)	1.13 (0-2)
Q4 People in the surrounding of the child can take proper care in case of an allergic reaction?	2.28 (0-6)	1.5 (1-2)

Numbers indicate: 0= never; 1= very small chance; 2= small chance; 3= reasonable chance; 4=likely; 5 very likely; 6100% sure; 9= unknown.
